# Comparative analyses of the faecal resistome against β-lactam and quinolone antibiotics in humans and livestock using metagenomic sequencing

**DOI:** 10.1038/s41598-023-48221-2

**Published:** 2023-11-28

**Authors:** Jieun Kim, Youna Cho, Suk-Kyung Lim, Mi-Ran Seo, Jang Won Sohn, Bongyoung Kim, Mina Rho, Hyunjoo Pai

**Affiliations:** 1 Department of Internal Medicine, College of Medicine, Hanyang University, Seoul, 04763 Republic of Korea; 2 Department of Computer Science and Engineering, Hanyang University, Seoul, 04763 Republic of Korea; 3 Bacterial Disease Division, Animal and Plant Quarantine Agency, 177 Hyeokin 8-ro, Gimcheon-si, Gyeongsangbuk-do 39660 Republic of Korea; 4 ConnectaGen Inc., F 203, MisaCentumbiz 2F, Jojeong-Dearo, Hanam-si, Gyeonggi-do 12918 Republic of Korea; 5 Department of Biomedical Informatics, Hanyang University, Seoul, 04763 Republic of Korea

**Keywords:** Computational biology and bioinformatics, Microbiology, Medical research

## Abstract

To assess the prevalence and abundance of antibiotic resistance genes in human and livestock gut microbiomes, 87 humans (healthy individuals and patients with *Clostridioides difficile* infection (CDI)) and 108 livestock (swine, cattle, and chickens) were enrolled. Gut microbiomes and fluoroquinolone-resistant *Escherichia coli* isolates were sequenced, and mobile genetic elements adjacent to the β-lactamase (*bla*) and transferable quinolone resistance (*qnr*) genes were compared using metagenomic contigs. Each group of humans and livestock exhibited distinctive microbiota and resistome compositions in the gut. Concerning the resistome of *bla* and *qnr*, the prevalence rates between chickens and patients with CDI were the most similar (R^2^ = 0.46); *bla*_TEM_, *bla*_OXA_, *bla*_CTX-M_, and *qnrS* were highly prevalent in both groups. According to genomic and phylogenetic analyses, *bla*_CTX-M_ and *bla*_OXA_ expressed lineage specificity to either humans or livestock, while *qnrS* and *bla*_TEM_ displayed a shared lineage between humans and livestock. A *qnrS1* mobilome comprising five genes, including two recombinases, a transposase, and a plasmid gene, is commonly found in human and chicken gut microbiomes. Humans and chickens showed the most similar gut resistomes to β-lactams and quinolones. *QnrS* and *bla*_TEM_ displayed especially strong co-occurrence between the guts of humans and livestock.

## Introduction

The increased use of antibiotics in both humans and livestock has promoted the spread of antibiotic resistance genes (ARGs) worldwide^1–3^. The use of antimicrobials in livestock exerts selection pressure for propagation in animals with antimicrobial resistance (AMR) microbes, which are occasionally human pathogens^4^. Moreover, findings suggesting the transfer of ARGs between bacteria of human and animal origins have been reported, and horizontal gene transfer plays a greater role than the clonal spread of strains between these niches^4,5^.

In human medicine, β-lactam and quinolone antibiotics accounted for over 50% and 10% of the prescribed antibiotics, respectively^6^. Therefore, major β-lactamase families mediated by mobile genetic elements, such as extended-spectrum β-lactamases (ESBLs), plasmid-mediated AmpC cephalosporinases (pAmpCs), and carbapenemases, are particularly concerning. ESBL-positive *Escherichia coli* has become widespread in community infections, and particularly, the intestinal tracts of animals represent essential reservoirs for ESBL-producing *E. coli*^7^. Transferable quinolone resistance genes, such as *qnr*, are disseminated in the gut of humans and animals and are related to various mobile genetic elements, including plasmids, transposons, genomic islands, and other specific antibiotic resistance determinants^8^.

Previous studies presenting shared features of resistance genes, plasmids, or clones among animal and human sources have usually compared *Enterobacteriaceae* isolates^9–11^. In recent years, metagenomic sequencing technology has enabled the analysis of the arrangement of specific genes and mobilomes without cultivating isolates^12–14^, providing an effective tool for investigating ARG transmission mechanisms in natural environments. In a study characterizing interconnections involving human faeces and the environment, resistomes and phylogenetic composition were tightly linked, whereas key resistance genes crossed habitat boundaries and were associated with mobile genetic elements^15^.

In this study, we investigated the similarity and distribution of ARGs between food animals and humans residing in an urban setting with little direct contact with living livestock. Using bacterial genomes and metagenomes sequenced from the gut microbiomes of healthy individuals, patients with *Clostridioides difficile* infection (CDI), chickens, swine, and cattle, the abundance, prevalence, and arrangement of ARGs and their mobilome structures were studied. Our results indicated that the distributions of ARGs against β-lactams and fluoroquinolones in the gut microbiome were most similar between CDI patients and chickens, and the *bla*_TEM_, *bla*_CTX-M_, *bla*_*OXA*_, and *qnrS* genes exhibited a strong tendency to co-occur between humans and livestock.

## Results

### Distribution of bacteria and ARGs in humans and livestock

The composition of bacteria and ARGs were compared between human and livestock guts. For humans, we included two groups: 61 healthy individuals and 26 CDI patients, who were expected to show an augmented ARG profile in the gut microbiome. For livestock, 36 cattle, 41 swine, and 31 chickens were included because they produce a large proportion of edible commodities.

**Figure 1:**
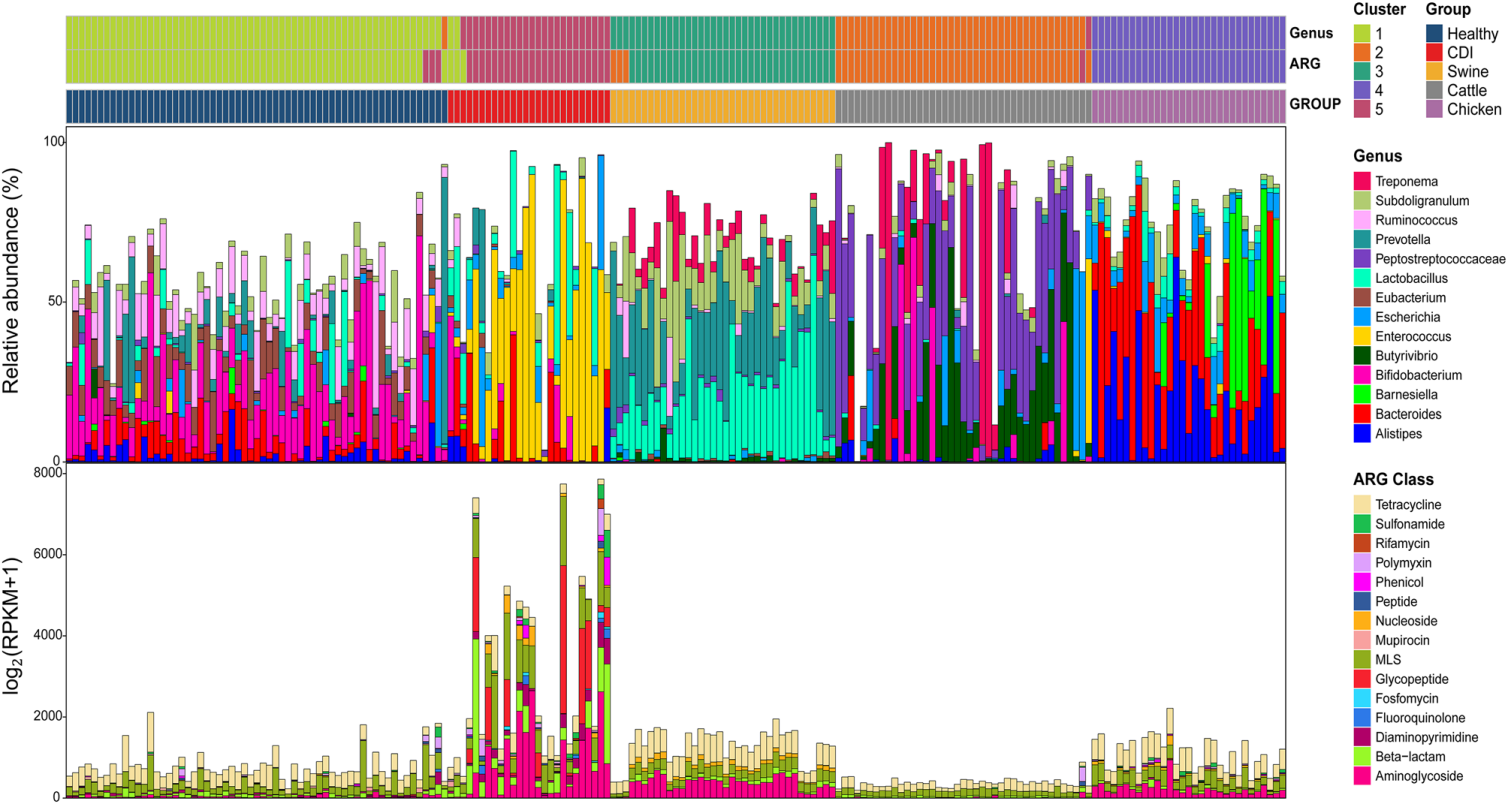
Characterization of the microbiome and antibiotic resistance genes (ARGs) in each group. The two bars at the top of the plot represent the result of Dirichlet multinomial mixture (DMM) clustering based on genus and ARG compositions, respectively. The relative bacterial composition abundance was plotted for a set of genera that ranked in the top three in any group. For the ARG class, the cumulative relative abundance of ARGs in each class was visualized.

Each group exhibited a distinct microbial composition (*p* = 0 and *p* = 0, respectively; Wilks’ lambda test; Fig. 1 and Supplementary Table S1). When the samples were clustered using the DMM model, the optimal Laplace approximation value was five, where most of the samples in each group were clustered together (at the top in Fig. 1; Supplementary Fig. S1A,B). Each group presented different dominant genera in their gut: *Bifidobacterium*, *Ruminococcus*, and *Bacteroides* in healthy people; *Enterococcus*, *Lactobacillus*, and *Bacteroides* in CDI patients; *Prevotella*, *Lactobacillus*, and *Subdoligranulum* in swine; *Peptostreptococcus*, *Butyrivibrio*, and *Treponema* in cattle; and *Bacteroides*, *Alistipes*, and *Barnesiella* in chickens.

The ARG distribution also showed different patterns between the groups (Fig. 1). When the samples were clustered by the relative abundance of ARGs, a similar pattern to that based on the microbial composition was observed, and most of the samples in each group were located close together and assigned to the same cluster when the DMM algorithm was applied (at the top in Fig. 1; Supplementary Fig. S1C,D). An average of 0.07% of the reads in each sample were aligned to the ARG sequences. CDI patients exhibited fourfold higher ARG abundances than healthy individuals (3347.9 vs. 792 RPKM in Table 1), while cattle harboured the fewest ARGs, followed by chickens and swine, among the livestock (378.4 vs. 1103.5 vs. 1281.6). Humans were found to have more diverse ARGs than livestock, and chickens harboured the most diverse ARGs and cattle the least among livestock (105 vs. 81; the number of different ARGs; Table 1). However, when the prevalence of existing ARGs was compared, chickens showed the highest prevalence, followed by CDI patients (24.6% vs. 23.0%; the average prevalence of existing ARGs; Table 1).

**Table 1:** Statistics of antibiotic resistance genes (ARGs) in healthy individuals, patients with *Clostridioides difficile* infections, swine, cattle, and chickens.

	Healthy	CDI	Swine	Cattle	Chickens
Sum of average RPKM values for all ARGs	792.01	3347.89	1281.60	378.35	1103.47
Number of different ARGs	109	152	82	81	105
The average prevalence of existing ARGs (%)	12.86	22.97	17.39	9.54	24.65

RPKMs, reads per kilobase of transcript per million mapped reads.

### Prevalence of β-lactamase and transferable quinolone resistance genes

**Figure 2:**
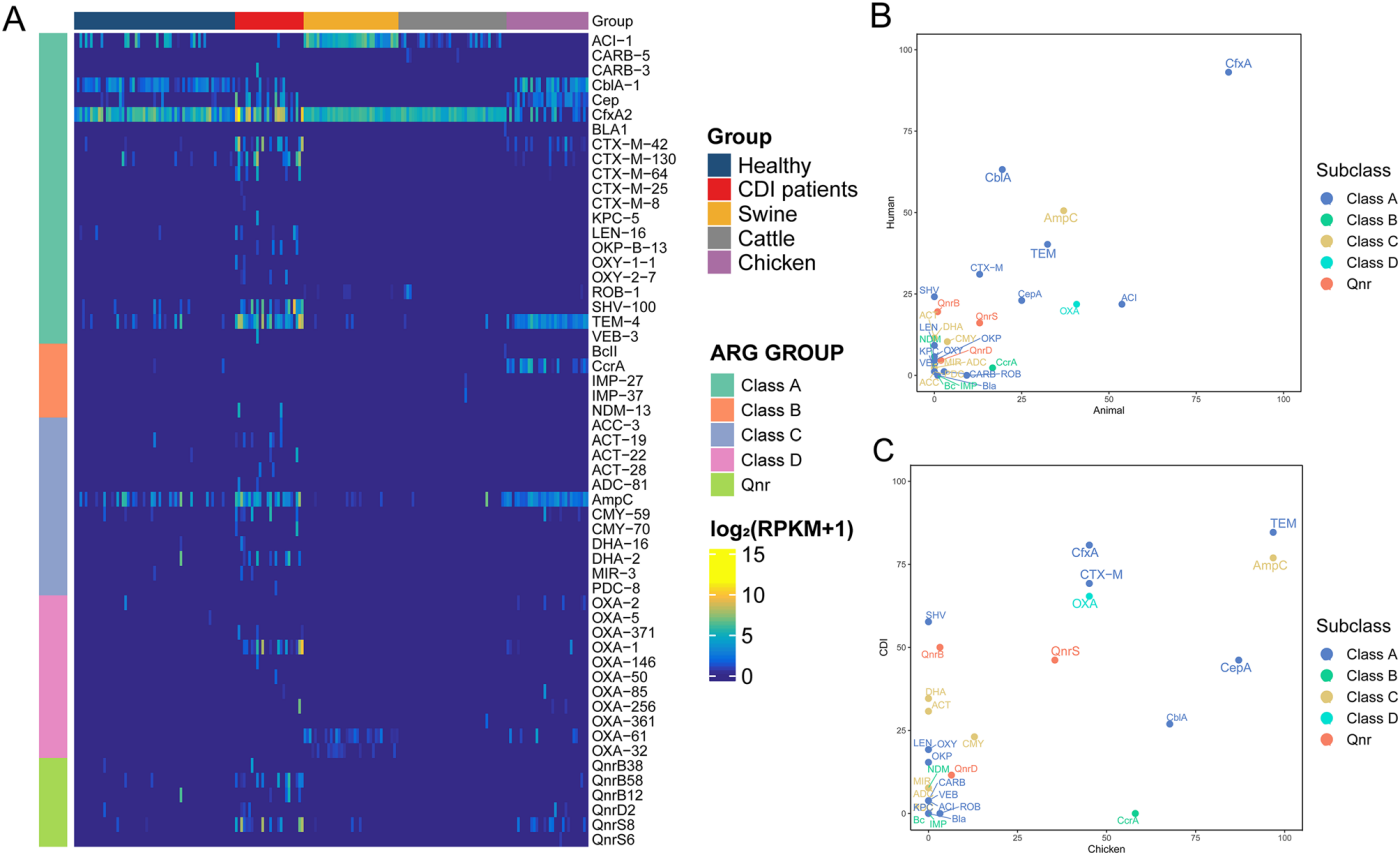
Abundance and prevalence of β-lactamase and transferable quinolone resistance genes. (**A**) Relative abundance of β-lactamase and transferable quinolone resistance genes in each sample, measured by reads per kilobase of transcript per million mapped reads (RPKM). (**B**) Prevalence of β-lactamase and transferable quinolone resistance gene families in humans and livestock. (**C**) Prevalence of β-lactamase and transferable quinolone resistance gene families in patients and chickens with *Clostridioides difficile* infection (CDI). The names of highly prevalent ARGs (> 30%) in both groups are enlarged.

In humans, the abundance of most ARGs against β-lactams, quinolones, aminoglycosides, MLS, and glycopeptides was significantly higher in patients with CDI than in healthy individuals (*p* < 0.0005, Student’s *t* test; Figs. 1 and 2; Supplementary Tables S2 and S3). Among β-lactam resistance genes (Supplementary Table S4), *bla*_CTX-M_ and *bla*_TEM_ showed significant differences (0.73 vs. 51.76 and 1.44 vs. 59.50 RPKM for healthy individuals vs. CDI patients; *p* < 0.0001 for each Student’s *t* test; Supplementary Tables S2 and S3). More than half of the patients with CDI had these genes (Supplementary Table S3).

When the prevalence of β-lactamases and transferable quinolone resistance genes was compared between humans and each group of livestock, seven genes, *ampC*, *bla*_TEM_, *bla*_OXA,_ *bla*_CTX-M_, *cfxA, cepA*, and *qnrS*, were prevalent in both CDI patients and chickens (prevalence in both groups > 30%) but were rarely found in cattle or swine (Fig. 2A, Supplementary Table S3).

Among the plasmid-associated β-lactamase genes, *bla*_TEM_ and *bla*_OXA_ were prevalent in both humans and livestock (40.2% vs. 32.4% for *bla*_TEM_ and 21.8% vs. 40.7% for *bla*_OXA_; Fig. 2B,C, and Supplementary Table S3). *bla*_TEM_ was the most prevalent β-lactamase gene in CDI patients and chickens (84.6% and 96.8%, respectively) but not in swine or cattle (8.3% and 4.9%, respectively). *blaOXA* prevalence was high in CDI patients, chickens, and swine (69.2%, 45.2, and 80.6%, respectively). However, *bla*_SHV_ was distributed exclusively in the human gut (9.8% in healthy adults and 57.7% in patients with CDI vs. 0% in cattle, swine, and chickens).

Among the ESBL genes, *bla*_CTX-M_ was widely distributed in CDI patients and chickens (65.4% and 45.2%, respectively) but was not identified in swine or cattle (Fig. 2B,C, and Supplementary Table S3). Most *pAmpC* genes (*bla*_DHA_*, bla*_ACT_, *bla*_MIR_, *and bla*_ACC_) were exclusively observed in human samples, whereas *bla*_CMY_ was detected in healthy humans, patients with CDI, and chickens (4.9%, 23.8%, and 12.9%, respectively). The carbapenemase genes *bla*_KPC_ and *bla*_NDM_ were not observed in these animals. One *bla*_IMP_-containing cattle sample was identified.

In terms of chromosomal β-lactamase genes, *cfxA* was the most common β-lactamase gene present in anaerobic organisms, found in both human and animal guts, followed by *cblA* and *cepA* (Supplementary Tables S2 and S3)*.* These genes exist in the chromosomes of *Bacteroides* and *Prevotella*, which are the major constituents of human and livestock guts. *AmpC* has been frequently detected in CDI patients and chickens, suggesting enrichment of the phylum Proteobacteria, especially Enterobacteriaceae^16,17^.

Concerning transferable quinolone resistance genes, *qnrS* was prevalent in CDI patients and chickens but was rarely found in swine and cattle (46.15% and 35.48% of CDI patients and chickens, respectively; 8.33% and 0% of swine and cattle, respectively; Fig. 2 and Supplementary Table S3). Particularly, we found that 16 of the 26 patients with CDI (61.5%) had one or more *qnr* genes. Fourteen chicken samples (45.2%) contained *qnr* genes, with *qnrS* (35.48%) being the most prevalent family. The distribution of *qnrS* exhibited a statistically significant difference between the different livestock groups (*p* < 0.01 for swine vs. chickens and cattle vs. chickens; Student’s *t* test); *qnrS* was found in only three swine samples (8.3% prevalence) and was not identified in any of the cattle samples.

### Genomic analysis of co-occurring *bla* and *qnr* genes in humans and livestock

**Figure 3:**
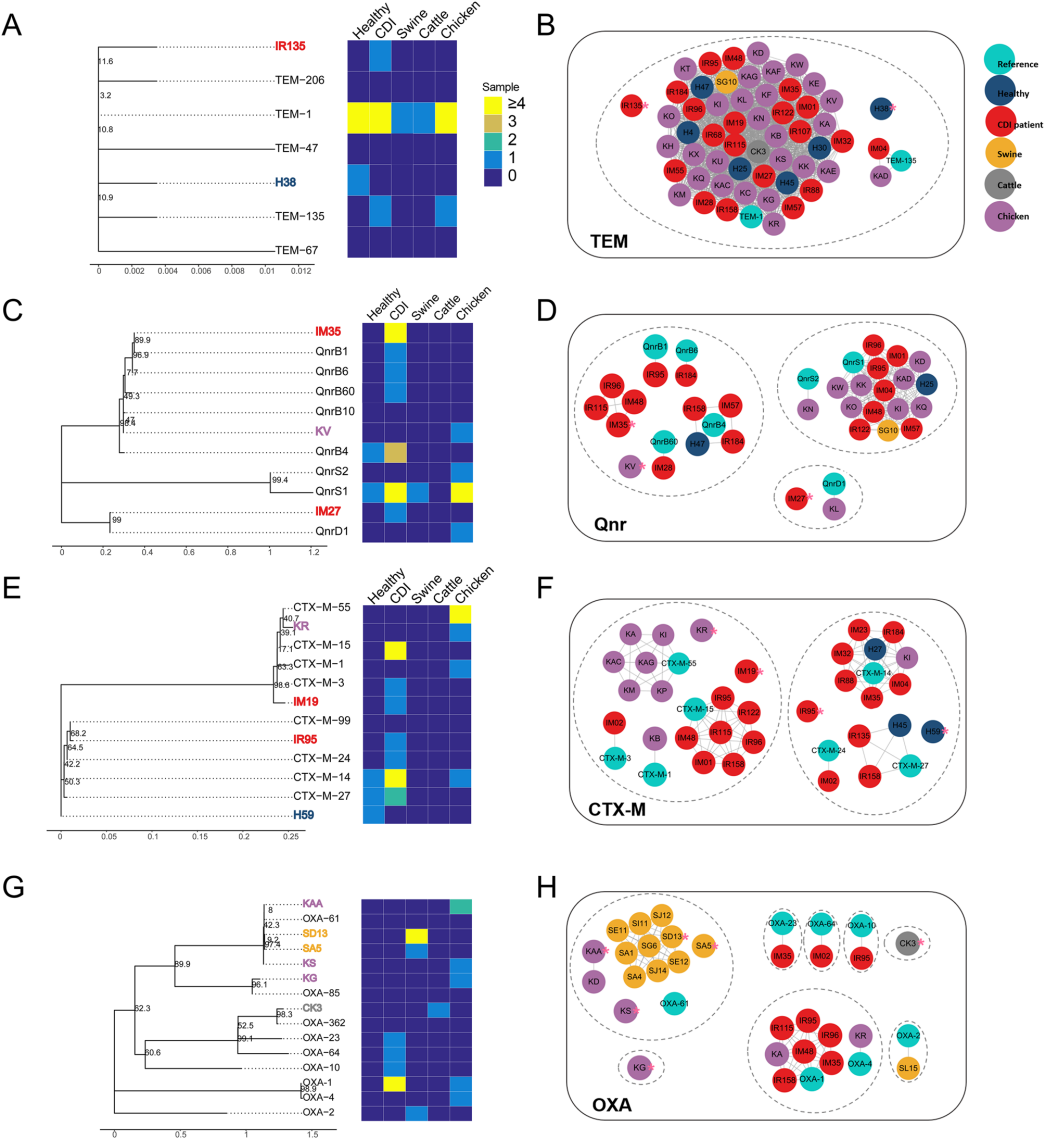
Homology and phylogenetic relationships of *bla*_TEM_, *qnr*, *bla*_CTX-M_, and *bla*_OXA_ genes. Phylogenetic tree and prevalence of (**A**) *bla*_TEM_, (**C**) *qnr*, (**E**) *bla*_CTX-M_, and (**G**) *bla*_OXA_ genes. The name in black is the reference sequence. The names in other colours represent sequences obtained from specific samples, whose colours indicate the group. The coloured node is a novel sequence similar to the reference sequence. The gene sequence was obtained from the metagenomic assembly. Network of (**B**) *bla*_TEM_, (**D**) *qnr*, (**F**) *bla*_CTX-M_, and (**H**) *bla*_OXA_ genes. A node represents a sequence found in the given sample. The colour represents the host group. Nodes are connected with a solid line when both sequences are identical. Nodes are clustered with a dotted line when they have a similarity above 95%. Cytoscape (v.3.8, https://cytoscape.org) was used to build network graphs.

To investigate the genomic sequences and phylogenetic relationships involving *bla* and *qnr* genes among different hosts, the complete *bla*_CTX-M_, *bla*_OXA_, *bla*_TEM_, and *qnrS* genes were obtained through metagenomic assembly (Fig. 3). In the network of ARGs, two sequence homology levels were investigated: identical genes were linked together as a connected component, and homologous (similarity ≥ 95%) ARG clusters were constructed (Fig. 3B,D,F,H). In the trees, ARG sequences obtained from each identical sequence group were compared to identify their phylogenetic relationships (Fig. 3A,C,E,G), revealing interesting patterns in which the *qnrS* and *bla*_TEM_ genes belonged to common lineages in humans and livestock. Specifically, *qnrS1* and *bla*_TEM_ were prevalent in both the human and livestock microbiomes.

The *bla*_TEM_ genes were clustered to construct a very large group of *bla*_TEM-1_ genes, which were identical to the known *bla*_TEM-1_ gene and mostly from humans and chickens (identical genes are connected with solid edges in Fig. 3B). As a minor group, two genes that were identical to *bla*_TEM-135_ were identified in one human and one chicken sample, and there was a gene that was one amino acid different from the *bla*_TEM-1_ gene. Homologous *bla*_TEM_ genes were clustered closely in the phylogenetic tree, as indicated by the distance at the bottom of the tree (Fig. 3A). Among the *qnr* genes, *qnrS* was the most prevalent. Most of the *qnrS* genes were *qnrS1,* commonly found in humans and chickens (Fig. 3D), and showed a pattern similar to that of *bla*_TEM_. In contrast, *qnrB* was primarily derived from humans.

### Co-occurrence of the ARG-carrying mobilome in humans and livestock

**Figure 4:**
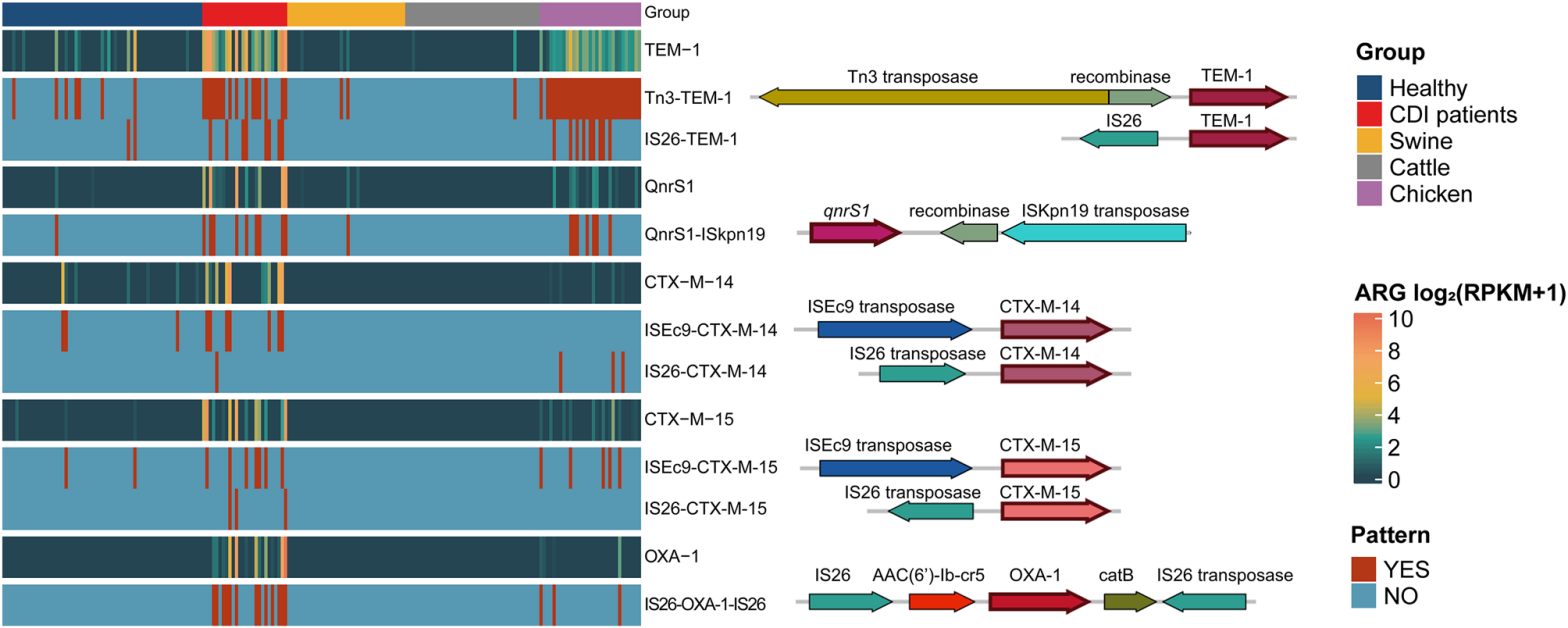
Dominant mobilomes related to the *bla*_TEM_, *qnr*, *bla*_CTX-M_, and *bla*_OXA_ genes. For each antibiotic resistance gene (ARG), the cell in the heatmap represents ARG abundance measured by (${\mathrm{log}}\_2\, ({\mathrm{RPKM}}+1)$). The abundance was measured by read mapping against the ARG sequences. The cell indicates whether the mobilome pattern exists in each sample for each mobilome. The pattern was named using the insertion sequence or transposase name with the ARG. The structure of each pattern is visualized next to its prevalence heatmap.

The ARG-carrying mobilomes for *bla*_TEM_, *bla*_CTX-M_, *bla*_OXA_, and *qnrS* were identified and compared between humans and livestock (Fig. 4 and Supplementary Table S5). Similar to the results from the ARG sequence analysis, a dominant mobilome structure for *bla*_TEM_ and *qnrS* was commonly found in humans and chickens. In all groups, *bla*_TEM-1_ with Tn3 (i.e., *Tn3- bla*_TEM-1_ pattern) was the most common and was found in 80% of *bla*_TEM_-carrying samples. In particular, 65.7% of *bla*_TEM_-carrying humans and 100% of *bla*_TEM_-carrying chickens showed the *Tn3-bla*_TEM-1_ pattern. The second most prevalent mobilome pattern for *bla*_TEM_ was *IS26- bla*_TEM_*,* which was found in healthy individuals (15.4%), patients with CDI (36.4%), and chickens (30.0%). For *qnrS1*, the dominant mobilome pattern was *qnrS1-ISkpn19*, which was found in 71.4% of *qnrS1*-carrying humans and 70.0% of *qnrS1*-carrying chickens (Fig. 4 and Supplementary Table S5). This observation implies that this mobilome pattern is efficient for ARG transmission in humans and chickens (see the next section for details on the *qnrS1-ISkpn19* mobilome structure).

For *bla*_CTX-M-14_ transmission, *ISEc9-bla*_CTX-M-14_ is a more common mobilome structure than *IS26* transposase in humans (i.e., *IS26-bla*_CTX-M-14_). For example, 45.5% of *bla*_CTX-M-14_ was carried by *ISEc9-bla*_CTX-M-14,_ and 18.2% was carried by *IS26-bla*_CTX-M-14_. In chickens, all *bla*_CTX-M-14_ genes were in the structure of *IS26-bla*_CTX-M-14_, and *ISEc9-bla*_CTX-M-14_ was not found. *Bla*_OXA-1_ was the most prevalent in patients with CDI, where 92.9% of CDI patients harbouring *bla*_OXA-1_ carried IS26-*bla*_OXA-1_-IS26, and three-quarters of the chickens carried it.

### High conservation of ARG-carrying mobilomes in bacterial genomes and metagenomes

**Figure 5:**
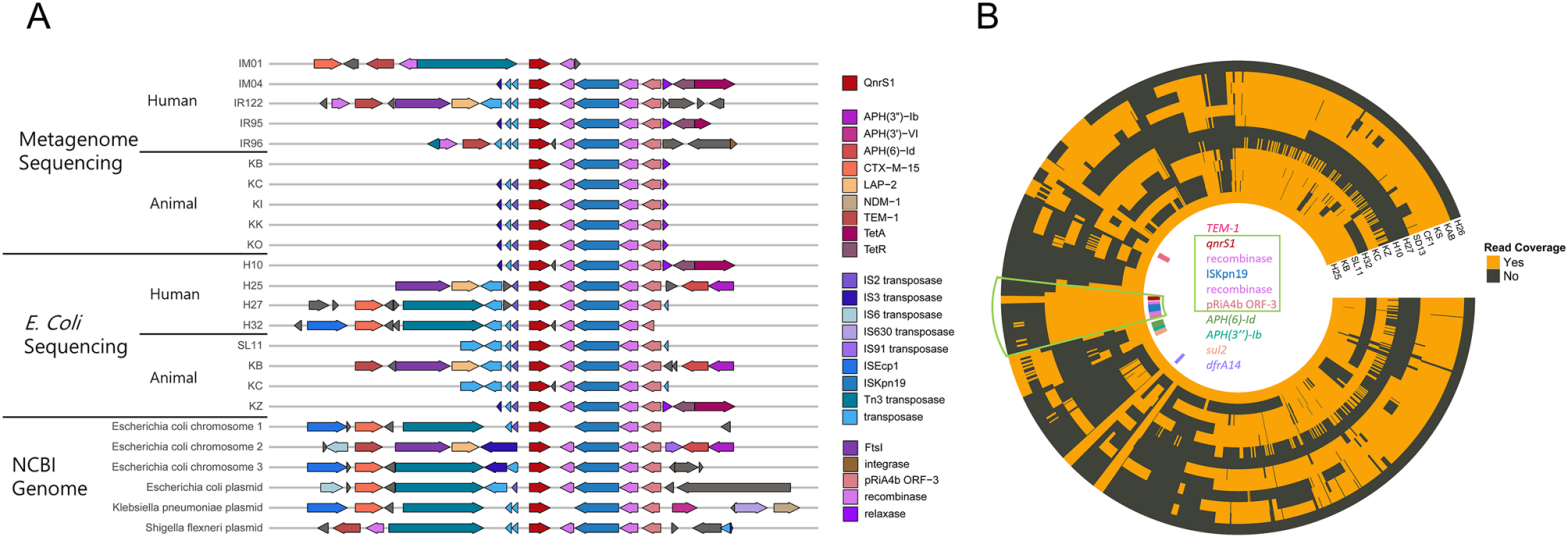
Arrangement of *qnrS* and its neighbouring genes. (**A**) *QnrS* and its adjacent genes were identified from metagenome assemblies, *E. coli* genome assemblies, and reference genomes in the NCBI repository. (**B**) Using the *E. coli* plasmid containing *qnrS1* as a reference, the regions mapped by using 15 *E. coli* isolates resistant to fluoroquinolone are coloured orange. *QnrS1*, its mobilome, and other ARGs in the plasmid are annotated with coloured rectangles in the centre.

Because *qnrS* exhibited a high rate of co-occurrence in humans and livestock (Fig. 4), mobilome structures were further characterized for this ARG using metagenomic contigs, *E. coli* genomes, and plasmid genomes. From the metagenome contigs, *qnrS1* genes were identified along with upstream and downstream genes in ten human and livestock samples. From the *E. coli* isolates sequenced in this study, *qnrS1* was identified in eight samples from humans and livestock. In the bacterial and plasmid genomes obtained from the NCBI repository, *qnrS1* genes were found in three *E. coli* chromosomes and three plasmids of *E. coli*, *Klebsiella pneumoniae*, and *Shigella flexneri* (Fig. 5A and Supplementary Table S6). Notably, most *qnrS1*-carrying mobilomes were composed of genes with the same pattern, including the *qnrS1* gene, a recombinase, the *ISKra4* family transposase, a recombinase, and a plasmid-related gene (Fig. 5A). These four neighbouring genes and *qnrS1* were well conserved, ranging from 99 to 100% in sequence similarity. Many transposases of the insertion sequence were observed in the regions upstream and downstream of *qnrS1* (Supplementary Table S7). *Tn3* family transposases were frequently found, and other ARGs, such as *bla*_CTX-M-15_, *bla*_TEM-1_, *lap-2*, *aph(6)-Id*, *aph(3″)-Ib*, and *sul2*, were found close to *qnrS1*.

Additionally, gene arrangements upstream and downstream of the *qnrS*-carrying mobilome in *E. coli* isolates were further investigated using sequencing reads. Using a complete plasmid sequence from sample H25 as a reference, the sequencing reads of 13 *E. coli* isolates with fluoroquinolone resistance were aligned to confirm that the gene arrangements upstream and downstream of *qnrS1*-carrying mobilomes were different among the *E. coli* isolates, even though *qnrS1*-carrying mobilomes were well conserved (Fig. 5B). Moreover, it was confirmed that nine *qnrS1-*containing isolates showed a well-conserved mobilome pattern of *qnrS1*, whereas the other four, which contained *qnrS2*, did not show the same mobilome pattern. We analysed chromosome multi-locus sequence typing (MLST) types and plasmid incompatibility groups for these *E. coli* isolates and found that all 13 *E. coli* isolates bearing the *qnrS* gene had different sequence types (STs), and 12 STs were previously found (Table 2). No specific association was found between chromosomal phylogenetic groups or plasmid incompatibility groups and the type of *qnrS* gene.

**Table 2:** Characterization of QnrS-carrying *E. coli* isolates and plasmids.

Sample	Group	Phylogenetic group	Plasmid incompatibility group	Sequence type (ST)	Qnr gene
KB	Chicken	A	IncF	NA	QnrS1
H32	Healthy	A	IncF	ST167	QnrS1
KC	Chicken	A	IncF and IncH12	ST2542	QnrS1
KZ	Chicken	A	NA	ST2207	QnrS1
H26	Healthy	A	NA	ST216	QnrS2
CF1	Cattle	B1	IncF	ST2179	QnrS2
H25	Healthy	B1	IncF	ST101	QnrS1
KS	Chicken	B1	IncF	ST2607	QnrS2
SL11	Swine	B1	IncF and Incl1	ST224	QnrS1
H10	Healthy	B2	NA	ST2346	QnrS1
KAB	Chicken	D	IncF	ST117	QnrS2
SD13	Swine	D	IncF	ST1034	QnrS1
H27	Healthy	D	IncF and IncH12	ST2797	QnrS1

## Discussion

In modern megacities, the food chain is considered a carrier of resistant bacteria or to enable possible horizontal gene transfer between humans and livestock^5,7,8,10,11,18^. The number of foodborne outbreaks associated with fresh vegetables has increased, and contamination by animal waste is mostly driven by improperly composted animal manure, water, and soil^19^. Retail meat, especially poultry meat, serves as a vehicle for human exposure and infection with highly resistant *E. coli*^20^. These findings highlight the relevance of antibiotic use in animal food production to antibiotic resistance in humans.

In this study, the similarities between ARGs in humans and livestock were studied, and mobile genetic elements adjacent to resistance genes were compared.

The results revealed that the overall distribution differed between humans and livestock. Interestingly, the PCoA based only on β-lactamase and *qnr* genes exhibited a different distribution pattern: patients with CDI and chickens were closely related. This suggests that both harbour many ARGs against β-lactams and fluoroquinolones and that their common ARGs are carried by the same types of mobile genetic elements. In the Republic of Korea, penicillin (22.7%) and enrofloxacin (18.2%) are the best-selling antibiotics for chicken breeding, followed by phenicols, ionophores, and tetracyclines^21^. The high prevalence of *bla*_CTX-M_ in chicken gut bacteria, despite the low usage of extended-spectrum cephalosporins (ESCs) in breeding, might be because the *qnrS*, *bla*_TEM_, and *bla*_CTX-M_ genes are frequently located in the same plasmid or in any other mobile element and co-transferred^11,22^.

*bla*_TEM_, *bla*_OXA_, *bla*_CTX-M_, and *qnrS* were the most prevalent β-lactamase or transferable quinolone resistance genes in the guts of humans and livestock and were also found in similar genetic contexts. In contrast, several ARGs, such as *bla*_SHV_, *bla*_DHA_, *bla*_ACT_, *bla*_MIR_, and *qnrB,* have been observed in the human gut, although some were observed in livestock in other studies^9,23,24^. The prevalence of these genes in the gut of healthy humans was not as high as that of *bla*_TEM_ or *bla*_CTX-M_, which reside in human and livestock guts. Among *pAmpC* family members, *bla*_CMY_ was identified in both patients with CDI and livestock in this study, was prevalent in healthy humans as well, and is also known to be a predominant *pAmpC* in the animal sector, especially in poultry^2^. These findings suggest that an enlarged pool of ARGs carried by livestock may contribute to the prevalence of these genes in human pathogens.

Although we selected a cohort of humans and livestock to study the similarity of ARGs and analysed 195 metagenomes from humans and three livestock species in this study, the number of samples might not be sufficient to represent each host species, but we will continuously expand the size of the cohort and perform comparative studies. The sampling and sequencing protocols were carefully designed to maintain compatibility between different groups of samples.

In conclusion, the level and distribution of β-lactamase and *qnr* genes were the most similar between humans and chickens. Chickens had the highest abundance of β-lactam and fluoroquinolone ARGs, followed by pigs and cattle. *bla*_TEM_, *bla*_OXA_, *bla*_CTX-M_, and *qnrS* exhibited distinct features of co-occurrence in humans and non-human animals. Additionally, humans, chickens, and swine contain homologous *qnrS* gene cassettes in different plasmids.

## Materials and methods

### Study design and description of specimens

A total of 195 metagenomic samples were used: 61 from healthy individuals from Korea, 26 from patients with CDI, 36 from swine, 41 from cattle, and 31 from chickens (Table 3). Healthy individuals were defined as those scoring zero for the Charlson comorbidity index and having no hospital admission history within the past year^25^. Patients with CDI were selected to represent a human group with high ARG levels in their gut because of current or recent antibiotic usage^26^. Human samples were collected from Hanyang Medical Centers (HMC) located in Seoul and Guri, which is a satellite city connected to Seoul. Healthy individuals were all sampled from the health examination centre of HMC in Seoul, and 6 CDI patients were hospitalized in HMC in Seoul, but 20 CDI patients were enrolled from HMC in Guri. The individuals in this study had limited direct contact with food animals. The human participants were all from the Seoul area, while the livestock samples were collected from various locations across the Korean Peninsula. Sequencing data from healthy individuals, CDI patients, swine, and cattle were obtained in our previous studies^26,27^. Chicken faecal samples were collected and sequenced in this study. For the chicken microbiome, 155 faecal samples were collected from 31 farms (5 chickens from each farm) in the Republic of Korea, and five specimens from each farm were pooled for metagenomic sequencing. A total of 13 *E. coli* isolates from 5 humans, 2 swine, 1 cow, and 5 chickens were selected on MacConkey agar plates containing 0.25 or 0.5 μg/mL ciprofloxacin and were confirmed to have *qnrS* by *qnrS*-specific PCR^28^.

**Table 3:** Characteristics of gut microbiome samples from humans, cattle, swine, and chickens.

Group	Characteristics	Location	Year	Reference
Healthy adults (n = 61)	Zero scores for the Charlson comorbidity index No admission history within the past year Age between 30 and 59 years	Seoul, Korea	2017	^28^
Patients with CDI (n = 26)	Diarrhoea (> 3 stools/day) Toxigenic culture of *C. difficile* or A and B toxin assay (VIDAS^®^ *C. difficile* Toxin A and B; BioMerieux SA, Marcy l’Etoile, France) was positive	2 Hanyang Univ. Hosp. located in Seoul and Guri, Korea	2016–2018	^28^
Cattle (n = 41)	Collected from 8 farms located in 6 provinces in the Republic of Korea	Korea	2018	^29^
Swine (n = 36)	Collected from 7 farms located in 6 provinces in the Republic of Korea	Korea	2017	^29^
Chickens (n = 31)	155 faeces samples were collected from 31 farms (5 chickens on each farm) in the Republic of Korea	Korea	2019	This study

CDI, *Clostridioides difficile* infection.

### DNA preparation

Each faecal sample was thoroughly homogenized using a spatula and divided into 250–300 mg aliquots. Total DNA was extracted using the Fast DNA SPIN Kit for Feces (MP Biomedicals, Santa Ana, CA, USA) following the manufacturer’s instructions.

Genomic DNAs of 13 *E. coli* isolates were extracted from overnight cultures using the QIAamp DNA Micro Kit (Qiagen, Hilden, Germany) following the manufacturer’s instructions.

### Sequencing and processing of reads

All microbiome samples were sequenced on the Illumina HiSeq platform. For each sample, all reads were 151-bp paired-end sequences with an insert size of ~ 350 bp. Quality trimming was performed using Sickle (v.1.33)^29^ with Phred quality scores > 20 and read length options > 90 bp (pe -q 20 -t sanger -l 90). The reads that aligned to their corresponding host genome using Bowtie2-align (v.2.1.0)^30^ with the sensitive-local option were removed. The host reference genomes for humans, swine, cattle, and chickens were downloaded from the NCBI repository (accession numbers: GCA_000001405.1, GCF_000003025.6, GCF_000003055.6, and GCF_000002315.6, respectively). After quality trimming, an average of 91.7% of the sequence reads were conserved.

Thirteen *E. coli* isolates with fluoroquinolone resistance were sequenced using the Illumina HiSeqX platform. One *E. coli* isolate from a healthy adult, H25, was sequenced on the PacBio platform to obtain high-quality complete genome sequences.

### Composition analysis of microbial communities

The microbiota composition was determined using MetaPhlAn 2.0^31^. To minimize noise, genera with a < 0.1% abundance were not used for the analysis. The genus composition was used for the Dirichlet multinomial mixture (DMM) analysis^32^. This approach constructs a frequency matrix of the given taxa for each sample and builds a probabilistic model. Figure 1 shows the compositions of the top three most abundant genera in each group.

### Profiling ARG abundance and prevalence

To profile ARGs, an ARG reference database was established from 2252 sequences in CARD (v.2.0.1)^33^. To reduce redundant mapping, homologous sequences were clustered using cd-hit (v.4.7) (-c 0.9 -n 8)^34^, resulting in 848 representative ARG sequences.

To measure ARG abundance, reads were mapped using Bowtie2 (v2.3.4.1) (sensitive-local). Reads per kilobase of transcript per million mapped reads (RPKM) values were used to measure the abundance of ARGs in the given samples. Only reads that met the following criteria were used for RPKM calculations: > 50% of the read length was aligned against an ARG sequence and had a similarity of > 90%. The ARG was considered to be present only when 70% of the length of an ARG was covered by one of the mapped reads. The number of samples containing a specific ARG was counted to measure the prevalence of ARGs in each group.

ARG analysis was conducted using gene, family, or class measures. The ARG family is a cluster of ARGs (e.g., *qnrS1*, *qnrS2*, and seven other genes belonging to the *qnrS* family), and the ARG class is a cluster of ARG families that confer resistance to the same class of antibiotics (e.g., *qnrS*, *qnrB*, and *qnrD* belonging to the fluoroquinolone class).

### Identification of complete ARGs and sequence analysis

A three-step procedure was performed to predict the complete ARGs and their neighbouring genes. First, the reads were assembled using MEGAHIT (v.1.1.3) (default option)^35^. Second, gene prediction was carried out using FragGeneScan (v.1.31) using only contigs > 500 bp^36^, with the option of no sequencing errors (-w 1 -t complete). Finally, genes were identified as ARGs using BLASTp (v.2.6.0+)^37^ against the ARG database with an e-value threshold of 1 × 10^−10^, a similarity > 90%, and a reference coverage > 70%.

All applicable ARGs were visualized for network analysis. Pairwise similarities between the ARGs were measured using BLAST (v2.7.1+). Identical ARGs were clustered together by solid lines. A representative ARG was selected from each cluster if the cluster did not have any annotated ARGs obtained from the CARD database. Phylogenetic analysis was performed using the selected representative ARGs and annotated ARGs from the CARD database. First, multiple sequence alignments were performed on the amino acid sequences of ARGs using Muscle^38^. Phylogenetic distance was calculated along the branches of the tree using megacc^39^ with the following options (Jones–Taylor–Thornton (JTT) model; partial deletion = 95%; bootstrap value = 50). The phylogenetic tree was visualized using a heatmap from the ggtree library.

### Identification of ARG-carrying mobilomes

Contigs harbouring ARGs and neighbouring genes were analysed to define the patterns of ARG-carrying mobilomes using the NCBI database. Two conditions had to be satisfied to confirm the existence of a mobilome in each microbiome: > 70% of the reference mobilome was mapped by sample reads, and one or more paired-end reads (i.e., conjunction reads) were aligned in both the ARG and the neighbouring gene. Conjunction reads were defined on the basis of the following: (i) > 20% of each read and aligned to either the ARG or its neighbouring gene, (ii) both paired reads with < 10 bp of soft clips, which are the regions at the end of reads and are not aligned to the reference sequence, and (iii) the distance between paired reads < 350 × 3 bp.

### Analysis of *qnrS* gene arrangements and neighbouring genes

To analyse *qnrS* and neighbouring genes, three different types of genomic sequences were used: (i) contigs assembled from the microbiome, (ii) contigs assembled from *E. coli* isolates, and (iii) *E. coli* chromosomes and plasmid sequences obtained from the NCBI repository. A circular coverage map was constructed by mapping the sequencing reads of *E. coli* isolates to fully assembled sequences of *E. coli* plasmids containing *qnrS* genes.

The 13 PMQR gene-carrying *E. coli* isolates were characterized. The phylogenetic group was determined by identifying the presence of the chuA, yjaA, and TspE4.C2 genes^40^ using blastn (similarity and coverage ≥ 90%). For the plasmid incompatibility group, the reference locus database was downloaded from PubMLST (https://pubmlst.org/). Plasmids were categorized according to the presence of a specific locus (similarity and coverage ≥ 90%). For example, SD13 was categorized as IncF because it contained the FII_95 and FIB_13 loci. Finally, the sequence type (ST) was annotated using seven ST-related genes (adk, fumC, gyrB, icd, mdh, purA, and recA) downloaded from EnteroBase (https://enterobase.warwick.ac.uk/). The ST number was determined using the seven ST-related genes.

When plotting BRIG, the *E. coli* plasmid H25, which was sequenced with the PacBio long-read sequencing platform, was used as a reference. The total length of the plasmid genome is 97,230 bp. The samples used as a query were 12 *E. coli* plasmid isolates sequenced by the Illumina HiSeqX platform. The option used for Blast was 1e−10.

### Statistical analyses

Bray‒Curtis dissimilarity was measured when performing the principal coordinate analysis (PCoA) using the cmdscale function in the stats library. The clusters for microbial and ARG families were determined using DMM with the dmn function in the Dirichlet multinomial package. The optimal number of clusters was chosen using the Laplace index. To compare the distributions of ARGs between the two groups, a two-tailed Student’s *t* test was performed using SciPy**.** Statistical significance was set at* P* < 0.05.

### Ethics approval and consent to participate

The study was conducted according to the guidelines of the Declaration of Helsinki. The study protocol was approved by the Institutional Review Board of Hanyang University Hospital (HYUH IRB 2017-06-001 and HYUH IRB 2016-05-031), and written informed consent was obtained from all the participants. All methods were carried out in accordance with relevant guidelines and regulations.

### Supplementary Information

Supplementary Tables.

Supplementary Figures.

## Data Availability

Whole-genome sequencing data of *E. coli* in this study are available from the European Nucleotide Archive (ENA) under accession number PRJEB38960. Gut microbiome sequencing data of healthy individuals, CDI patients, swine, cattle, and chickens are available from the ENA under accession numbers PRJEB38960, PRJEB33013, PRJEB35738, PRJEB32496, PRJEB38606, and PRJEB38606.
