# Supplementary material for: Comparative analyses of the faecal resistome against β-lactam and quinolone antibiotics in humans and livestock using metagenomic sequencing
Source: Sci Rep. 2023 Nov 28;13:20993. doi: 10.1038/s41598-023-48221-2 (PMC10684531; doi:10.1038/s41598-023-48221-2)
Supplement: Supplementary file 1 — Supplementary Tables. [file 41598_2023_48221_MOESM1_ESM.docx]

Supplementary Table S1. Average relative abundance of bacteria at the genus level in the microbiome of healthy individuals, patients of *Clostridioides difficile* infection, swine, cattle, chickens

| Genus  relative abundance (%) | Healthy human | CDI patients | Swine | Cattle | Chickens |
| --- | --- | --- | --- | --- | --- |
| Acidaminococcus | 0.03 | 0 | 0.40 | 0 | 0 |
| Acinetobacter | 0 | 0.06 | 0.01 | 3.33 | 0 |
| Actinomyces | 0.03 | 0.10 | 0 | 0 | 0 |
| Adlercreutzia | 0.50 | 0 | 0 | 0 | 0 |
| Akkermansia | 1.58 | 3.41 | 0 | 0.10 | 1.63 |
| Alistipes | 2.21 | 1.51 | 0.02 | 1.04 | 19.74 |
| Anaerostipes | 0.55 | 0.16 | 0 | 0 | 0 |
| Anaerotruncus | 0 | 0.01 | 0 | 0 | 0.71 |
| Arthrobacter | 0 | 0 | 0 | 1.59 | 0 |
| Bacillus | 0 | 0 | 0 | 0.25 | 0 |
| Bacteroidales_noname | 0.11 | 0 | 0 | 0 | 1.18 |
| Bacteroides | 5.53 | 7.50 | 0.04 | 0.85 | 29.11 |
| Barnesiella | 0.36 | 0.07 | 0.03 | 0 | 11.82 |
| Bifidobacterium | 16.15 | 3.37 | 0.07 | 6.19 | 0 |
| Bilophila | 0.19 | 0.28 | 0 | 0 | 2.10 |
| Blautia | 4.20 | 3.32 | 0.22 | 0.10 | 2.46 |
| Brachybacterium | 0 | 0 | 0.02 | 0.35 | 0 |
| Brevibacterium | 0 | 0 | 0.11 | 0.19 | 0 |
| Butyricicoccus | 0 | 0.23 | 0 | 0 | 0.63 |
| Butyrivibrio | 0.17 | 0 | 0.96 | 16.75 | 0 |
| Campylobacter | 0 | 0 | 0 | 0 | 0.78 |
| Candida | 0 | 0.14 | 0 | 0 | 0 |
| Catenibacterium | 1.71 | 0 | 0.72 | 0 | 0 |
| Citrobacter | 0.02 | 0.39 | 0 | 0 | 0 |
| Clostridiales_noname | 0.11 | 0.10 | 0 | 0 | 0 |
| Clostridium | 0.46 | 2.01 | 0 | 0.43 | 0.78 |
| Collinsella | 4.07 | 0.22 | 0.07 | 0 | 0 |
| Coprobacillus | 0.19 | 0.79 | 0 | 0 | 0 |
| Coprobacter | 0.01 | 0 | 0 | 0 | 0.22 |
| Coprococcus | 2.16 | 0 | 2.96 | 0 | 0 |
| Corynebacterium | 0 | 0.16 | 0.33 | 0.37 | 0 |
| Dermabacteraceae_unclassified | 0 | 0 | 0 | 0.21 | 0 |
| Desulfovibrio | 0.01 | 0.14 | 0.55 | 0 | 0 |
| Dialister | 2.22 | 0.11 | 0.10 | 0 | 0 |
| Dietzia | 0 | 0 | 0 | 0.76 | 0 |
| Dorea | 3.49 | 0.04 | 0.55 | 0.02 | 0 |
| Eggerthella | 0.54 | 0.58 | 0 | 0 | 0 |
| Enterobacter | 0.04 | 0.85 | 0 | 0 | 0 |
| Enterococcus | 0.43 | 36.20 | 0 | 1.46 | 0.45 |
| Erysipelotrichaceae_noname | 2.26 | 1.39 | 0.56 | 0.56 | 0.93 |
| Escherichia | 1.77 | 5.18 | 0.48 | 5.63 | 7.02 |
| Eubacterium | 7.89 | 0.51 | 0.44 | 0 | 0 |
| Faecalibacterium | 7.73 | 0.26 | 2.18 | 0 | 0.04 |
| Fusobacterium | 0 | 0.10 | 0 | 0 | 0 |
| Haemophilus | 0.20 | 0.16 | 0 | 0 | 0 |
| Halomonas | 0 | 0 | 0 | 0.50 | 0 |
| Helicobacter | 0 | 0 | 0.40 | 0 | 0.58 |
| Klebsiella | 0.12 | 3.37 | 0.06 | 0 | 0 |
| Lachnospiraceae_noname | 1.91 | 0.64 | 0.02 | 0.30 | 0.27 |
| Lactobacillus | 3.14 | 9.52 | 22.41 | 0.24 | 3.26 |
| Leuconostoc | 0.11 | 0.18 | 0 | 0 | 0 |
| Marinobacter | 0 | 0 | 0 | 0.13 | 0 |
| Megamonas | 3.60 | 0 | 0 | 0 | 0 |
| Megasphaera | 0.19 | 0.10 | 2.34 | 0.01 | 0 |
| Mitsuokella | 0.22 | 0 | 3.02 | 0.02 | 0 |
| Morganella | 0 | 0.32 | 0 | 0 | 0 |
| Odoribacter | 0.12 | 0.11 | 0 | 0.25 | 1.61 |
| Olsenella | 0.01 | 0.03 | 0 | 0.21 | 0 |
| Oscillibacter | 0.12 | 0.04 | 0.83 | 0.47 | 3.19 |
| Parabacteroides | 0.68 | 2.07 | 0.77 | 3.61 | 6.09 |
| Paraprevotella | 0.28 | 0 | 0 | 0 | 0 |
| Parvimonas | 0 | 0.11 | 0 | 0.15 | 0 |
| Pediococcus | 0.02 | 1.33 | 0.01 | 0.01 | 0 |
| Peptostreptococcaceae_noname | 0.45 | 0.51 | 0.99 | 27.68 | 0.03 |
| Peptostreptococcus | 0 | 0.28 | 0 | 0 | 0 |
| Phascolarctobacterium | 0.33 | 0 | 4.96 | 0 | 0 |
| Porphyromonas | 0 | 0.14 | 0 | 0 | 0 |
| Prevotella | 5.52 | 1.97 | 23.15 | 0.17 | 0 |
| Proteus | 0 | 0.18 | 0 | 0 | 0.06 |
| Pseudoflavonifractor | 0 | 0 | 0 | 0 | 0.93 |
| Pseudomonas | 0 | 0.02 | 0.06 | 2.96 | 0 |
| Pusillimonas | 0 | 0 | 0 | 0.13 | 0 |
| Roseburia | 1.10 | 0.02 | 0.21 | 0.36 | 0 |
| Ruminococcaceae_noname | 0 | 0 | 0.25 | 0 | 0 |
| Ruminococcus | 8.71 | 0.49 | 1.78 | 0.97 | 0 |
| Selenomonas | 0 | 0 | 7.88 | 0.02 | 0 |
| Staphylococcus | 0 | 0.50 | 0.08 | 0 | 0 |
| Streptococcus | 1.73 | 2.55 | 0.04 | 2.70 | 0 |
| Streptomyces | 0 | 0 | 0 | 0.22 | 0 |
| Subdoligranulum | 3.44 | 1.41 | 12.58 | 1.98 | 3.91 |
| Succinivibrionaceae_unclassified | 0 | 0 | 0.15 | 3.09 | 0 |
| Sutterellaceae_unclassified | 0.02 | 0 | 0.53 | 0 | 0.04 |
| Treponema | 0 | 0 | 6.18 | 12.77 | 0 |
| Veillonella | 0.19 | 3.15 | 0 | 0 | 0 |
| Weissella | 0.24 | 0.16 | 0 | 0 | 0 |
| Yaniella | 0 | 0 | 0 | 0.27 | 0 |

CDI, *Clostridioides difficile* infection

Supplementary Table S2. Average abundance of β-lactamase and plasmid-mediated quinolone resistance genes in healthy individuals, patients with *Clostridioides difficile* infection, swine, cattle, chickens

| RPKM | β-lactamase &  PMQR Subclass | β-lactamase &  PMQR genes | Healthy human | CDI patients | Swine | Cattle | Chickens |  |
| --- | --- | --- | --- | --- | --- | --- | --- | --- |
|  |  |  |  |  |  |  |  |  |
| Beta-lactam | Class A | *ACI* | 5.99 | 0.11 | 37.59 | 1.42 | 0 |  |
|  |  | *Bla* | 0 | 0 | 0 | 0.02 | 0 |  |
|  |  | *CARB* | 0 | 0.83 | 0 | 0.05 | 0 |  |
|  |  | *CblA* | 3.97 | 1.21 | 0 | 0 | 9.33 |  |
|  |  | *cepA* | 0.31 | 9.07 | 0 | 0 | 3.37 |  |
|  |  | *CfxA* | 38.58 | 245.17 | 40.09 | 35.01 | 7.39 |  |
|  |  | *CTX-M* | 0.73 | 51.76 | 0 | 0 | 0.65 |  |
|  |  | *KPC* | 0 | 0.44 | 0 | 0 | 0 |  |
|  |  | *LEN* | 0.04 | 0.78 | 0 | 0 | 0 |  |
|  |  | *OKP* | 0 | 0.38 | 0 | 0 | 0 |  |
|  |  | *OXY* | 0 | 0.45 | 0 | 0 | 0 |  |
|  |  | *ROB* | 0 | 0 | 0.04 | 0.22 | 0.01 |  |
|  |  | *SHV* | 0.20 | 37.35 | 0 | 0 | 0 |  |
|  |  | *TEM* | 1.44 | 59.80 | 0.07 | 0.12 | 6.69 |  |
|  |  | *VEB* | 0 | 0.69 | 0 | 0 | 0 |  |
|  | Class B | *Bc* | 0 | 0 | 0 | 0.01 | 0 |  |
|  |  | *CcrA* | 0.03 | 0 | 0 | 0 | 4.50 |  |
|  |  | *IMP* | 0 | 0 | 0 | 0.07 | 0 |  |
|  |  | *NDM* | 0 | 1.53 | 0 | 0 | 0 |  |
|  | Class C | *ACC* | 0 | 0.06 | 0 | 0 | 0 |  |
|  |  | *ACT* | 0.08 | 1.36 | 0 | 0 | 0 |  |
|  |  | *ADC* | 0 | 0.23 | 0 | 0 | 0 |  |
|  |  | *ampC* | 4.52 | 20.40 | 0.08 | 2.63 | 4.63 |  |
|  |  | *CMY* | 0.04 | 3.68 | 0 | 0 | 0.35 |  |
|  |  | *DHA* | 1.47 | 3.33 | 0 | 0 | 0 |  |
|  |  | *MIR* | 0.01 | 0.09 | 0 | 0 | 0 |  |
|  |  | *PDC* | 0 | 0.05 | 0 | 0 | 0 |  |
|  | Class D | *OXA* | 0.10 | 56.47 | 1.15 | 0.05 | 1.36 |  |
| Fluoroquinolone | Qnr | *QnrB* | 1.16 | 5.11 | 0 | 0 | 0.02 |  |
|  |  | *QnrD* | 0.02 | 0.10 | 0 | 0 | 0.27 |  |
|  |  | *QnrS* | 0.02 | 29.93 | 0.06 | 0 | 0.59 |  |

^*^ Values in RPKM

CDI, *Clostridioides difficile* infection

Supplementary Table S3. Prevalence of β-lactamase and plasmid-mediated quinolone resistance genes in healthy individuals, patients with *Clostridioides difficile* infection, swine, cattle, chickens

| β-lactamase &  PMQR Subclass | β-lactamase &  PMQR genes | Healthy human (%) | CDI patients (%) | Swine (%) | Cattle (%) | Chickens (%) |  |
| --- | --- | --- | --- | --- | --- | --- | --- |
|  |  |  |  |  |  |  |  |
| *Class A* | *ACI* | 29.51 | 3.85 | 100 | 53.66 | 0 |  |
|  | *Bla* | 0 | 0 | 0 | 2.44 | 0 |  |
|  | *CARB* | 0 | 3.85 | 0 | 7.32 | 0 |  |
|  | *CblA* | 78.69 | 26.92 | 0 | 0 | 67.74 |  |
|  | *cepA* | 13.11 | 46.15 | 0 | 0 | 87.1 |  |
|  | *CfxA* | 98.36 | 80.77 | 100 | 100 | 45.16 |  |
|  | *CTX-M* | 14.75 | 69.23 | 0 | 0 | 45.16 |  |
|  | *KPC* | 0 | 3.85 | 0 | 0 | 0 |  |
|  | *LEN* | 4.92 | 19.23 | 0 | 0 | 0 |  |
|  | *OKP* | 0 | 15.38 | 0 | 0 | 0 |  |
|  | *OXY* | 0 | 19.23 | 0 | 0 | 0 |  |
|  | *ROB* | 0 | 0 | 16.67 | 7.32 | 3.23 |  |
|  | *SHV* | 9.84 | 57.69 | 0 | 0 | 0 |  |
|  | *TEM* | 21.31 | 84.62 | 8.33 | 4.88 | 96.77 |  |
|  | *VEB* | 0 | 3.85 | 0 | 0 | 0 |  |
| *Class B* | *Bc* | 0 | 0 | 0 | 2.44 | 0 |  |
|  | *CcrA* | 3.28 | 0 | 0 | 0 | 58.06 |  |
|  | *IMP* | 0 | 0 | 0 | 2.44 | 0 |  |
|  | *NDM* | 0 | 7.69 | 0 | 0 | 0 |  |
| *Class C* | *ACC* | 0 | 3.85 | 0 | 0 | 0 |  |
|  | *ACT* | 3.28 | 30.77 | 0 | 0 | 0 |  |
|  | *ADC* | 0 | 7.69 | 0 | 0 | 0 |  |
|  | *ampC* | 39.34 | 76.92 | 16.67 | 9.76 | 96.77 |  |
|  | *CMY* | 4.92 | 23.08 | 0 | 0 | 12.9 |  |
|  | *DHA* | 1.64 | 34.62 | 0 | 0 | 0 |  |
|  | *MIR* | 1.64 | 7.69 | 0 | 0 | 0 |  |
|  | *PDC* | 0 | 3.85 | 0 | 0 | 0 |  |
| *Class D* | *OXA* | 3.28 | 65.38 | 80.56 | 2.44 | 45.16 |  |
| *Qnr* | *QnrB* | 6.56 | 50 | 0 | 0 | 3.23 |  |
|  | *QnrD* | 1.64 | 11.54 | 0 | 0 | 6.45 |  |
|  | *QnrS* | 3.28 | 46.15 | 8.33 | 0 | 35.48 |  |

CDI, *Clostridioides difficile* infection

Supplementary Table S4. Statistical tests of abundance of antibiotic resistance genes (ARGs) between each paired groups (*p*-value)

| Subclass | Group1 | Healthy  individuals | Healthy  individuals | Healthy  individuals | Healthy  individuals | CDI  patients | CDI  patients | CDI  patients | Swine | Swine | Cattle | Human^2^ |
| --- | --- | --- | --- | --- | --- | --- | --- | --- | --- | --- | --- | --- |
|  | Group2 | CDI  patients | Swine | Cattle | Chicken | Swine | Cattle | Chicken | Cattle | Chicken | Chicken | Chicken |
| Class A | *ACI* | 0.029286 | 0 | 0.034987 | 0.015523 | 0 | 0.016665 | 0.278792 | 0 | 0 | 0.00446 | 0.044874 |
|  | *CblA* | 0.033123 | 0.000223 | 0.000085 | 0.010555 | 0.013761 | 0.008549 | 0.004056 |  | 0.0001 | 0.000034 | 0.000597 |
|  | *cepA* | 0.001306 | 0.217376 | 0.187896 | 0.000001 | 0.0103 | 0.006175 | 0.137912 |  | 0.000007 | 0.000002 | 0.838668 |
|  | *CfxA* | 0.005116 | 0.850137 | 0.632787 | 0.000414 | 0.031856 | 0.018971 | 0.021786 | 0.175058 | 0 | 0 | 0.109281 |
|  | *CTX-M* | 0.000054 | 0.372329 | 0.340838 | 0.928618 | 0.001583 | 0.000754 | 0.003749 |  | 0.045515 | 0.032748 | 0.131811 |
|  | *LEN* | 0.022235 | 0.251629 | 0.221003 | 0.287605 | 0.063297 | 0.047317 | 0.085125 |  |  |  | 0.29967 |
|  | *OKP* | 0.003484 |  |  |  | 0.025065 | 0.016756 | 0.03772 |  |  |  | 0.266275 |
|  | *OXY* | 0.005237 |  |  |  | 0.032321 | 0.022284 | 0.047152 |  |  |  | 0.286888 |
|  | *ROB* |  | 0.001185 | 0.113817 | 0.161879 | 0.033986 | 0.304036 | 0.364461 | 0.325486 | 0.071589 | 0.277202 | 0.094012 |
|  | *SHV* | 0.025507 | 0.16729 | 0.140458 | 0.20014 | 0.08563 | 0.066348 | 0.111075 |  |  |  | 0.38159 |
|  | *TEM* | 0.000068 | 0.269466 | 0.256684 | 0.002131 | 0.001648 | 0.000796 | 0.009021 | 0.69178 | 0.000004 | 0.000001 | 0.300976 |
| Class B | *CcrA* | 0.425199 | 0.347556 | 0.315905 | 0.000389 |  |  | 0.019523 |  | 0.006039 | 0.003399 | 0.000022 |
|  | *NDM* | 0.049955 |  |  |  | 0.133645 | 0.109078 | 0.164501 |  |  |  | 0.448844 |
| Class C | *ACT* | 0.023297 | 0.394439 | 0.363215 | 0.429634 | 0.061032 | 0.045426 | 0.082439 |  |  |  | 0.294267 |
|  | *ampC* | 0.00711 | 0.076022 | 0.540803 | 0.96782 | 0.002701 | 0.011505 | 0.029167 | 0.339561 | 0 | 0.494478 | 0.316581 |
|  | *CMY* | 0.006509 | 0.253078 | 0.222414 | 0.181868 | 0.035009 | 0.024372 | 0.081644 |  | 0.24496 | 0.214209 | 0.467026 |
|  | *DHA* | 0.466425 | 0.445238 | 0.415021 | 0.47896 | 0.036494 | 0.025534 | 0.052458 |  |  |  | 0.302683 |
| Class D | *OXA* | 0.015526 | 0.000275 | 0.673335 | 0.004253 | 0.068848 | 0.04758 | 0.092887 | 0.000773 | 0.749765 | 0.01176 | 0.390223 |
| Qnr | *qnrB* | 0.130572 | 0.430545 | 0.399983 | 0.474175 | 0.046937 | 0.033852 | 0.066709 |  | 0.284588 | 0.252957 | 0.250243 |
|  | *qnrS* | 0.001356 | 0.377386 | 0.34806 | 0.000079 | 0.014035 | 0.008621 | 0.025138 | 0.113682 | 0.004255 | 0.000626 | 0.256317 |

Twenty out of thirty-one ARGs, which showed p-values <0.05 between two samples, are presented at this table.

1. CDI, *Clostridioides difficile* infection
2. Human included healthy individuals and CDI patients

Supplementary Table S5. Mobilome ratio in Figure4.

| Mobilome Ratio (%) | Healthy human | CDI patient | Swine | Cattle | Chicken | Human | All group |
| --- | --- | --- | --- | --- | --- | --- | --- |
| Tn3-TEM-1 | 53.85 | 72.73 | 66.67 | 50 | 100 | 65.71 | 80.00 |
| IS26-TEM-1 | 15.38 | 36.36 | 0 | 0 | 30 | 28.57 | 27.14 |
| ISEc9-CTX-M-14 | 42.86 | 70 | 0 | 0 | 0 | 58.82 | 45.45 |
| IS26-CTX-M-14 | 0 | 10 | 0 | 0 | 60 | 5.88 | 18.18 |
| ISEc9-CTX-M-15 | 66.67 | 58.33 | 0 | 0 | 41.67 | 60.00 | 51.85 |
| IS26-CTX-M-15 | 0 | 25.00 | 0 | 0 | 0 | 20.00 | 11.11 |
| IS26-OXA-1-IS26 | 0 | 92.86 | 0 | 0 | 75.00 | 92.86 | 88.89 |
| QnrS1-ISKpn19 | 50 | 75.00 | 33.33 | 0 | 70 | 71.43 | 66.67 |

The numerator is the number of specific mobilome pattern found in each group, and the denominator is the number of ARG found in each group.

Supplementary Table S6. Reference genomes that contain *qnrS* in the NCBI repository

| **Name in Figure 3** | **NCBI accession no.** |
| --- | --- |
| *Escherichia_coli*_1_chromosome | CP010116.1 |
| *Escherichia_coli*_2_chromosome | CP019213.1 |
| *Escherichia_coli*_3_chromosome | CP018976.1 |
| *Klebsiella_pneumoniae*_plasmid | CP009115.1 |
| *Shigella_flexneri*_plasmid | CP012734.1 |
| *Escherichia_coli*_plasmid | CP018207.1 |

Supplementary Table S7. Gene annotations in Figure 5. Similarity above 90% was annotated as the same gene (except hypothetical genes).

| Sample | A list of genes in order |
| --- | --- |
| Metagenome_CDI_IM01 | CTX-M-15, tryptophan synthase subunit beta like protein, TEM-1, recombinase, Tn3 transposase, QnrS1, recombinase, hypothetical protein |
| Metagenome_CDI_IM04 | IS3 transposase, transposase, transposase, QnrS1, recombinase, ISKpn19, recombinase, pRiA4b ORF-3, relaxase, TetR, TetA |
| Metagenome_CDI_IR122 | hypothetical protein, recombinase, TEM-1, hypothetical protein, FtsI, LAP-2, transposase, transposase, transposase, QnrS1, recombinase, ISKpn19, recombinase, pRiA4b ORF-3, hypothetical protein, hypothetical protein, hypothetical protein, hypothetical protein |
| Metagenome_CDI_IR95 | IS3 transposase, transposase, transposase, QnrS1, recombinase, ISKpn19, recombinase, pRiA4b ORF-3, relaxase, TetR, TetA |
| Metagenome_CDI_IR96 | Tn3 transposase, recombinase, TEM-1, transposase, transposase, transposase, QnrS1, hypothetical protein, recombinase, ISKpn19, recombinase, pRiA4b ORF-3, UmuC, hypothetical protein, integrase |
| Metagenome_Chicken_KB | QnrS1, recombinase, ISKpn19, recombinase, pRiA4b ORF-3, relaxase, IS3 transposase, transposase, IS2 transposase, QnrS1, recombinase, ISKpn19, recombinase, pRiA4b ORF-3, relaxase |
| Metagenome_Chicken_KC | IS3 transposase, transposase, IS2 transposase, QnrS1, recombinase, ISKpn19, recombinase, pRiA4b ORF-3, relaxase |
| Metagenome_Chicken_KI | IS3 transposase, transposase, IS2 transposase, QnrS1, recombinase, ISKpn19, recombinase, pRiA4b ORF-3, relaxase |
| Metagenome_Chicken_KK | IS3 transposase, transposase, IS2 transposase, QnrS1, recombinase, ISKpn19, recombinase, pRiA4b ORF-3, relaxase |
| Metagenome_Chicken_KO | IS3 transposase, transposase, IS2 transposase, QnrS1, recombinase, ISKpn19, recombinase, pRiA4b ORF-3, relaxase |
| *E.coli_*Healthy_H10 | IS3 transposase, transposase, IS2 transposase, QnrS1, hypothetical protein, recombinase, ISKpn19, recombinase, pRiA4b ORF-3, relaxase, TetR, TetA |
| *E.coli*_Healthy_H25 | FtsI, LAP-2, transposase, hypothetical protein, IS2 transposase, QnrS1, recombinase, ISKpn19, recombinase, pRiA4b ORF-3, Zinc-binding protein, APH(6)-Id, APH(3'')-Ib |
| *E.coli*_Healthy_H27 | ParA, hypothetical protein, CTX-M-15, tryptophan synthase subunit beta like protein, hypothetical protein, Tn3 transposase, transposase, hypothetical protein, IS2 transposase, QnrS1, recombinase, ISKpn19, recombinase, pRiA4b ORF-3, transposase |
| *E.coli*_Healthy_H32 | hypothetical protein, ISEcp1, CTX-M-15, tryptophan synthase subunit beta like protein, hypothetical protein, Tn3 transposase, transposase, transposase, IS2 transposase, QnrS1, hypothetical protein, recombinase, ISKpn19, recombinase, pRiA4b ORF-3 |
| *E.coli_*Swine_SL11_ST224 | transposase, transposase, transposase, IS2 transposase, QnrS1, recombinase, ISKpn19, recombinase, pRiA4b ORF-3, transposase |
| *E.coli_*Chicken_KB_NA | TEM-1, FtsI, hypothetical protein, LAP-2, transposase, transposase, IS2 transposase, QnrS1, recombinase, ISKpn19, recombinase, pRiA4b ORF-3, hypothetical protein, hypothetical protein, APH(6)-Id, APH(3'')-Ib |
| *E.coli_*Chicken_KC_ST2542 | transposase, transposase, transposase, IS2 transposase, QnrS1, hypothetical protein, recombinase, ISKpn19, recombinase, pRiA4b ORF-3, transposase |
| *E.coli_*Chicken_KZ_ST2207 | IS3 transposase, transposase, IS2 transposase, QnrS1, recombinase, ISKpn19, recombinase, pRiA4b ORF-3, relaxase, TetR, TetA |
| *Escherichia coli* 1 chromosome | ISEcp1, hypothetical protein, CTX-M-15, tryptophan synthase subunit beta like protein, Tn3 transposase, transposase, IS2 transposase, QnrS1, ISKpn19, recombinase, pRiA4b ORF-3, phage tail protein |
| *Escherichia coli* 2 chromosome | hypothetical protein, IS6 transposase, TEM-1, FtsI, LAP-2, IS3 transposase, QnrS1, recombinase, ISKpn19, recombinase, pRiA4b ORF-3, IS91 transposase, APH(6)-Id, APH(3'')-Ib |
| *Escherichia coli* 3 chromosome | ISEcp1, hypothetical protein, CTX-M-15, tryptophan synthase subunit beta like protein, Tn3 transposase, IS3 transposase, transposase, QnrS1, recombinase, ISKpn19, recombinase, pRiA4b ORF-3, hypothetical protein, hypothetical protein, hypothetical protein |
| *Escherichia coli* plasmid | IS6 transposase, hypothetical protein, CTX-M-15, tryptophan synthase subunit beta like protein, Tn3 transposase, transposase, IS2 transposase, QnrS1, recombinase, ISKpn19, recombinase, pRiA4b ORF-3, hypothetical protein, hypothetical protein |
| *Klebsiella pneumoniae* plasmid | ISEcp1, CTX-M-15, tryptophan synthase subunit beta like protein, Tn3 transposase, transposase, transposase, QnrS1, ISKpn19, recombinase, pRiA4b ORF-3, APH(3')-VI, hypothetical protein, IS630 transposase, NDM-1 |
| *Shigella flexneri* plasmid | hypothetical protein, TEM-1, recombinase, Tn3 transposase, transposase, transposase, QnrS1, recombinase, ISKpn19, recombinase, pRiA4b ORF-3, hypothetical protein, hypothetical protein, transposase, IS3 transposase |

CDI, *Clostridioides difficile* infection
