# Supplementary material for: Comparative analyses of the faecal resistome against β-lactam and quinolone antibiotics in humans and livestock using metagenomic sequencing
Source: Sci Rep. 2023 Nov 28;13:20993. doi: 10.1038/s41598-023-48221-2 (PMC10684531; doi:10.1038/s41598-023-48221-2)
Supplement: Supplementary file 2 — Supplementary Figures. [file 41598_2023_48221_MOESM2_ESM.pptx]

## Slide 1
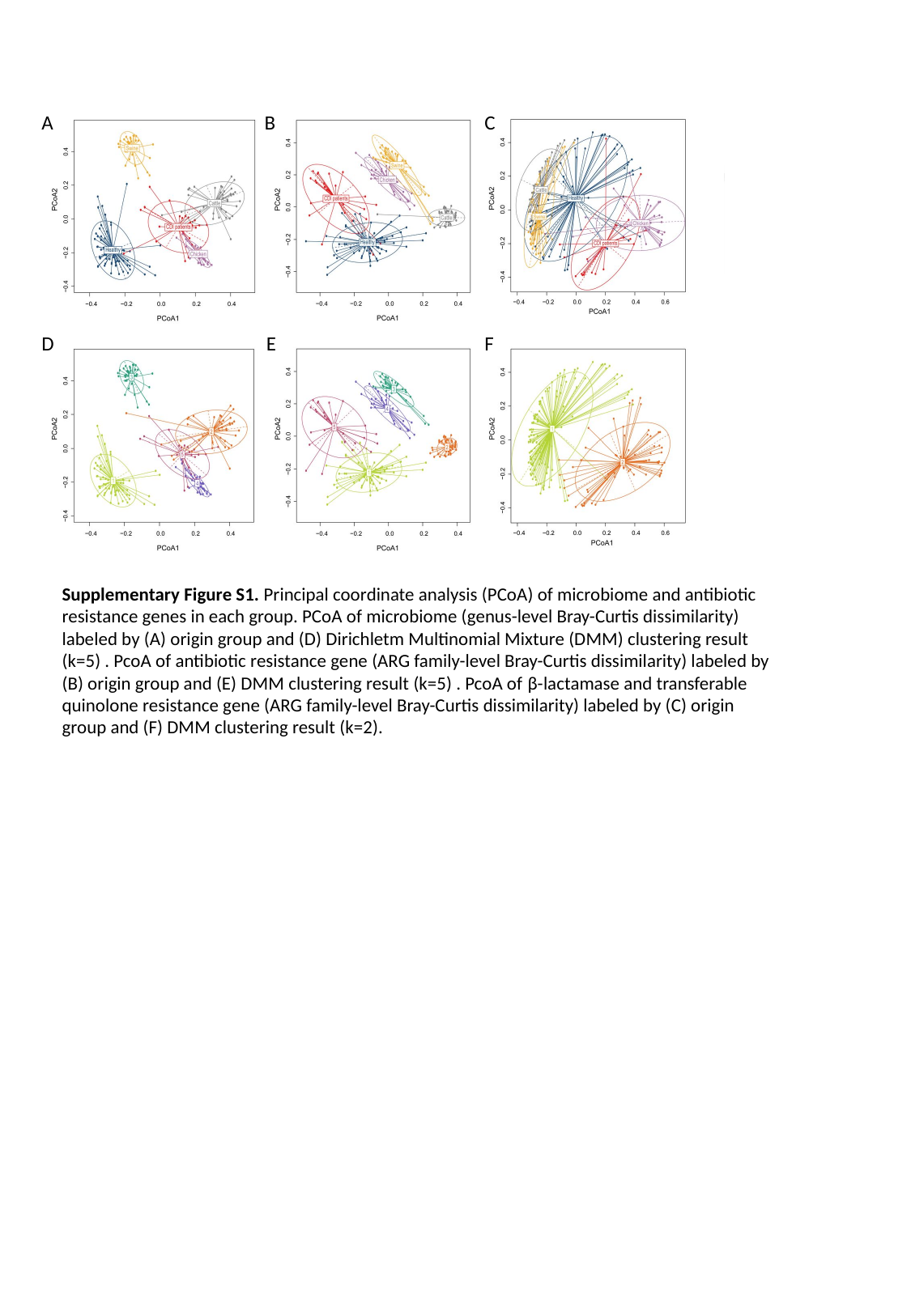

B
C
A
F
E
D
Supplementary Figure S1. Principal coordinate analysis (PCoA) of microbiome and antibiotic resistance genes in each group. PCoA of microbiome (genus-level Bray-Curtis dissimilarity) labeled by (A) origin group and (D) Dirichletm Multinomial Mixture (DMM) clustering result (k=5) . PcoA of antibiotic resistance gene (ARG family-level Bray-Curtis dissimilarity) labeled by (B) origin group and (E) DMM clustering result (k=5) . PcoA of β-lactamase and transferable quinolone resistance gene (ARG family-level Bray-Curtis dissimilarity) labeled by (C) origin group and (F) DMM clustering result (k=2).
